# A Comparison of Juvenile and Adult Sunflower Sea Star Transcriptomic Responses to Challenge With Sea Star Wasting Disease Tissue Homogenates

**DOI:** 10.1002/ece3.74379

**Published:** 2026-09-22

**Authors:** Grace Crandall, Alyssa‐Lois M. Gehman, Katherine Davis, Melanie B. Prentice, Drew Harvell, Samuel J. White, Paul Hershberger, Steven Roberts

**Affiliations:** ^1^ School of Aquatic and Fishery Sciences University of Washington Seattle Washington USA; ^2^ The Hakai Institute Campbell River British Columbia Canada; ^3^ Institute for the Oceans and Fisheries The University of British Columbia Vancouver British Columbia Canada; ^4^ Department of Earth, Ocean and Atmospheric Sciences The University of British Columbia Vancouver British Columbia Canada; ^5^ Washington Department of Fish and Wildlife Port Townsend Washington USA; ^6^ Department of Zoology The University of British Columbia Vancouver British Columbia Canada; ^7^ Friday Harbor Laboratories University of Washington Friday Harbor Washington USA; ^8^ Department of Ecology and Evolutionary Biology Cornell University Ithaca New York USA; ^9^ U.S. Geological Survey, Western Fisheries Research Center Marrowstone Marine Field Station Nordland Washington USA

**Keywords:** host immune response, *Pycnopodia helianthoides*, sea star wasting disease, sunflower sea star, transcriptomics

## Abstract

Sea star wasting disease (SSWD) has impacted over twenty sea star species along the Northeast Pacific Coast, with some seemingly more susceptible than others. The once common sunflower sea star, *Pycnopodia helianthoides* , has been driven to endangerment. Observations from natural SSWD outbreaks have indicated higher mortality rates in adults than juveniles, suggesting that susceptibility may be age‐dependent. The hypothesis of age‐related differences in immunity was assessed by challenging both juvenile and adult *P. helianthoides* in controlled laboratory exposures to unfiltered tissue homogenates prepared from SSWD‐affected adult *P. helianthoides* . Using a transcriptomic approach, we compared the immune response of adult and juvenile *P. helianthoides* exposed to SSWD in two independent disease challenge trials. In both trials, all disease‐exposed sea stars showed disease signs consistent with SSWD with no observable differences in outcome across age class, although age‐driven differentially expressed genes were identified, related to development, signaling, differentiation, and stress and metabolism regulation. Transcriptomic sequencing datasets showed a majority of differentially expressed genes were shared across the two experiments, suggesting a core response to SSWD that did not differ between age classes in the second trial. Functional annotation identified a core transcriptional response to challenge with grossly SSWD‐affected tissue homogenates, including genes annotated as having roles in defense response to bacterium.

## Graphical abstract

Sunflower sea stars have been hard hit by sea star wasting disease (SSWD), losing over 5 billion individuals along their natural range from Alaska and Southern California. This work uses transcriptomics to investigate the immune response of adult and juvenile sunflower sea stars exposed to SSWD. There is a conserved antibacterial response across the two age classes.

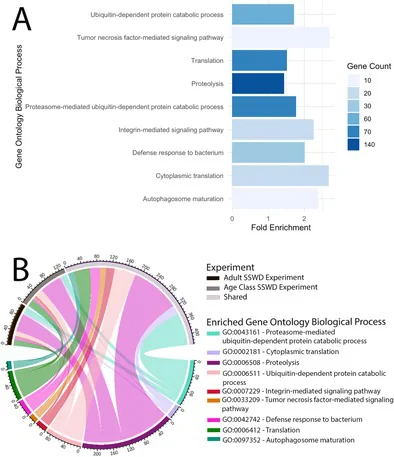

**Abbreviations**
ADA17: ADAM 17‐like protease
BIRC2: baculoviral IAP repeat‐containing protein 2
DHX15: DEAH‐box protein 15
IKKA: inhibitor of nuclear factor kappa‐B kinase subunit alpha
IKKB: inhibitor of nuclear factor kappa‐B kinase subunit beta
JNK: c‐Jun N‐terminal kinase
MAPK: mitogen‐activated protein kinase
MYD88: myeloid differentiation primary response 88
NF‐kB: nuclear factor kappa‐light‐chain‐enhancer of activated B cells
RGLG4: E3 ubiquitin‐protein ligase RGLG4 (RING domain ligase 4)
RIPK1: receptor‐interacting serine/threonine‐protein kinase 1
RNF31: ring finger protein 31
TN13B: tumor necrosis factor ligand superfamily member 13B
TNR19: tumor necrosis factor receptor superfamily member 19
TRAF2: tumor necrosis factor receptor‐associated factor 2
TRAF4: tumor necrosis factor receptor‐associated factor 4
TRAF5: tumor necrosis factor receptor‐associated factor 5
TRAF6: tumor necrosis factor receptor‐associated factor 6

## 1 Introduction

Understanding and managing wildlife disease outbreaks is a critical field of study as evidenced by the recent concerns around avian influenza and other zoonoses, which have impacted animal and human health alike (Krammer et al. 2025; Rahman et al. 2020). The health of foundation and keystone marine species and aquaculture species has been particularly impacted by increasing disease risks (Burge et al. 2014; Harvell et al. 2019; Harvell and Lamb 2020). Recent examples of ecosystem level impacts include disease‐driven mass mortality in seagrasses (Aoki et al. 2023), sea stars (Harvell et al. 2019), oysters (Raymond et al. 2022), corals (Alvarez‐Filip et al. 2022), and sea birds (Renner et al. 2024). Understanding disease outbreaks requires identification of causative agents and mechanisms of transmission. In parallel to this work, characterizing host immune responses is important as well because it can reveal mechanisms of resistance, identify candidate genes that distinguish susceptible from resilient individuals, and inform conservation and breeding strategies. Marine habitats are understudied, and although heavily impacted by recent mass mortalities, knowledge of innate immune mechanisms of marine invertebrates in particular is poorly understood.

One means to characterize host immune mechanisms is via transcriptomic sequencing, which can be used to identify and compare gene expression patterns in grossly diseased versus grossly healthy individuals, revealing clues to the molecular underpinnings of host responses to disease states. For example, in the commercially important flatfish, the turbot ( *Scophthalmus maximus* ), transcriptomics was used to investigate the differences in immune response between moderately and severely infected fish by a myxozoan parasite, revealing that while in early stages of infection, there aren't any significant changes in gene expression, but in more severe stages of infection, the genes related to the activation of the inflammatory response are differentially expressed, including interleukin 1b and interleukin 8 (Ronza et al. 2021). Transcriptomics can also be compared across host tissue types, thereby elucidating the role of different tissues in mounting an immune response to disease. Comparison of the mantle, gill, and adductor muscle in Korean mussels (*Mytilus unguiculatus*) exposed to the pathogen *Vibrio alginolyticus* found different roles that the tissue types play in immune response (Li et al. 2024). Transcriptomics can also reveal previously unknown responses to infections and novelties therein. For example, in the Pacific oyster, *Crassostrea gigas* , exposed to Ostreid herpesvirus 1 mVar (OsHV‐1mVar), transcriptomics revealed strong antiviral activity and some novel adaptors and receptors, like a novel toll‐like receptor and MYD88‐like genes (He et al. 2015).

Beyond characterizing the immune responses of hosts following infection with a known or unknown pathogen, transcriptomics can also offer valuable insights into the inter‐ and intra‐specific variation in these responses. For example, host susceptibility to disease can vary by life stage, which can impact management and conservation efforts. In juvenile (but not adult) Pacific oysters (*Crassostrea* [syn. *Magallana*] gigas), *Roseovarius crassostrea* sp. Nov. has caused mortality events in Maine, Massachusetts, and New York hatcheries (Boettcher et al. 2005). Similarly, infection with the bacteria *Vibrio pectenicida* is only known to occur in the larvae of several bivalve species (e.g., King scallops, *Pecten maximus* , and Pacific oysters) (Lambert et al. 1998). Host susceptibility to infection with Bd (*Batrachochytrium dendrobatidis*), which causes chytridiomycosis in Pacific treefrogs ( *Pseudacris regilla* ) and red legged frogs ( *Rana aurora* ), increases with age (Bradley et al. 2019). These examples highlight the nuances in host susceptibility and the specificity that some diseases have on certain life stages. Understanding the mechanisms underlying this variation can help to inform conservation practices through identifying and developing strategies to protect those life stages most at risk.

One of the largest disease outbreaks of marine wildlife is the ongoing Sea Star Wasting Disease (SSWD) epidemic that began in 2013 and extended from Mexico to Alaska. Sea star wasting has been observed for decades prior, however the outbreak starting in 2013 has been particularly disastrous, impacting over twenty sea star species (Dawson et al. 2023), with the most vulnerable being the sunflower sea star, *Pycnopodia helianthoides* , which has experienced losses of ~5.75 billion individuals (Heady et al. 2022) along their natural range from Southeast Alaska to Northern Baja, Mexico (Hamilton et al. 2021). The rapid loss of sunflower sea stars has resulted in the devastation of kelp forests, particularly in California where the species is functionally extirpated (Gravem et al. 2021; Hamilton et al. 2021). *Pycnopodia helianthoides* are keystone species that predate on sea urchins and reduce urchin damage on kelp forest habitats (Galloway et al. 2023). Kelp forests are important habitats for young fish and other animals, can act as carbon sinks, and the holdfasts can help prevent erosion. Restoring the urchin predator *P. helianthoides* to its natural range will thus help restore kelp forests. To aid in both kelp forest restoration and mitigate the drastic loss of *P. helianthoides* , conservation breeding efforts are underway and are looking to better understand the mechanisms of immune resistance in *P. helianthoides* . Conservation breeding can benefit from transcriptomic data informing strategies to increase immunity and disease resistance (Heady et al. 2022).

A bacterial causative agent of SSWD, *Vibrio pectenicida* strain FHCF‐3, has recently been identified (Prentice et al. 2025), and understanding the immune response of *P. helianthoides* to causative agent(s) of this disease remains underexplored. Sea stars have an innate immune response that involves both cellular and humoral responses. Cellularly, coelomocytes are the primary actors and are associated with processes like phagocytosis and production of antimicrobial peptides, among others, and there are several coelomocyte types, which vary among echinoderm groups (Smith et al. 2018). In addition to cellular responses, sea stars also exhibit humoral immune responses, including the production of proteins and other components that contribute to antipathogenic processes like opsonization and other pathways reviewed in Chiaramonte and Russo (2015). Understanding immune response following exposure to SSWD can help evaluate the potential for disease resistance in *P. helianthoides* .

Interestingly, early observations of SSWD in *P. helianthoides* reported many instances of adult *P. helianthoides* wasting, but rarely juveniles. Further, following SSWD disease emergence in the central coast of British Columbia, Canada, changes in the size distribution of *P. helianthoides* highlighted the loss of larger individuals (Gehman et al. 2025) suggesting that juveniles may be less affected. Age‐dependent disease trajectories have also been observed in other Pacific coast asteroids impacted by SSWD. In *Pisaster ochraceus* , adults exposed to SSWD showed disease signs more quickly than juveniles; however, once juveniles showed disease signs, they died more quickly than adults (Eisenlord et al. 2016). Together this suggests that SSWD could have varying effects across *P. helianthoides* life stages. Understanding if there is an age‐related change in immunity, and elucidating the underlying mechanisms of immunity can help inform management of this critically endangered keystone predator.

The goals of this study were to (1) characterize the immune response of *P. helianthoides* following experimental challenge with tissue homogenates prepared from a grossly SSWD‐affected adult, in which the recently described causative agent *Vibrio pectenicida* was subsequently detected, and (2) compare the susceptibility of adult versus juvenile *P. helianthoides* exposed to SSWD. Previous work studied the early stages of immune response in adult *P. helianthoides* (e.g., euthanized pre‐arm autotomy; Fuess et al. 2015). This study aimed to characterize the immune response of *P. helianthoides* in later stages of disease response following exposure.

To address the first goal, two disease challenge experiments were conducted and sea stars coelomocytes were sampled at the arm autotomy disease sign. Transcriptome datasets were generated to compare the gene expression response between grossly healthy and grossly SSWD affected specimens. Controlled disease challenge experiments can provide insights into the intricacies of immune response (Cardinaud et al. 2015; Gao et al. 2015; Yao et al. 2021), for example in this study, the immune response at disease signs of interest. The second goal was investigated via a disease challenge experiment which had two age classes, adults and juveniles, and sampled sea star coelomocytes at the arm autotomy disease sign for transcriptomic work to compare immune responses between age classes.

## 2 Methods

### 2.1 Animal Collection and Care

In the summer of 2021 and 2022, divers from the Washington Department of Fish and Wildlife (WDFW) collected adult (> 40 cm diameter) *P. helianthoides* from Dallas Bank off of Protection Island, WA (48.163165, −122.92805) and San Juan Island, WA (48.455, −123.016). These adults were used for two experimental trials referred to as “Adult SSWD Challenge” and “Age Class SSWD Challenge”, collected in 2021 and 2022, respectively (2021 WDFW Permit: 21‐1172; 2022 WDFW Permit: 22‐175). The adults for both years were collected at 50–60 ft depth in July and August (2021), and in May (2022). Juveniles (8–20 cm diameter) used in the Age Class SSWD Challenge were collected from Langley North on Whidbey Island, WA (48.0414, −122.4046), Baby Island South near Camano Island, WA (48.0908, −122.5266), and Langley Dock on Whidbey Island, WA (48.0385, −122.4018) (2022 WDFW Permit: 22‐175). The juveniles were collected at 35–45 ft with one collection at 63 ft in early June and in late July.

For the Adult SSWD Challenge (2021), adult sea stars were collected via SCUBA and placed in mesh bags, which were then hung from the stern of WDFW boats at marinas. Bags were collected shortly after SCUBA collection, and the sea stars were placed in coolers filled with ice and lined with seawater‐dampened cotton cloth. Sea stars were layered in the coolers and separated by layers of cloth to prevent physical contact between individuals. For the Age Class SSWD Challenge (2022), the same approach was taken for the adults and juveniles, except that sea stars were directly placed into coolers from SCUBA collection. Both of these experiments are part of the collection of experiments analyzed and discussed in Prentice et al. (2025).

Following collection, sea stars were transported to the USGS Marrowstone Field Station in Washington, Nordland, USA. Upon arrival, the sea stars were placed in individual tanks (37.8 L for adults and 5.8 L for juveniles) with individual tank flow‐through seawater (0.5 L/min) and individual air bubblers. The tanks were partially submerged in a water table in order to maintain a consistent temperature, with the outflow of each tank contributing to the shared water table outflow. In the facility, water was pumped from the ocean at a depth of 70 ft, sand filtered, UV treated, and filtered to 10 μm. The temperature was maintained at ~10.5**°**C using chillers. Since the potential for recent field exposure was unknown, individuals were monitored daily during a 2‐week quarantine period and those remaining grossly “normal” after this period were presumed healthy and used in experiments. Two weeks of quarantine provided enough time to observe any potential disease signs, as sea stars exposed in the field will have shown signs of wasting within this time frame. The status grossly normal refers to sea stars that were relaxed in appearance, with even turgor and no twisting of the arms. The sea stars were fed locally collected and subsequently frozen clams and mussels three times/week. Any uneaten clams and/or mussels were removed at the subsequent feeding and water quality was maintained due to the high flow rates in each tank.

### 2.2 Experimental Design

For the Adult SSWD Challenge, 16 quarantined adult *P. helianthoides* were selected and paired based on size (diameters ranged from 24 to 52 cm) (Data S1). One individual from each pair was randomly assigned to one of two treatment groups: (1) exposed (*n* = 8), which were injected with unfiltered raw tissue homogenate made from a wasting adult *P. helianthoides* , or (2) control (*n* = 8), which were injected with heat‐killed tissue homogenate made from the same wasting adult *P. helianthoides* (Table 1). Tissue homogenate inoculum was comprised of mixtures of tissue types from field‐collected wasting adult *P. helianthoides*, mixed using a Tissue Tearor Homogenizer; 220/240 VAC, 0.6 A (Cole‐Parmer, UX‐04750‐55), with seawater from the wasting sea stars' tank. The homogenate was then centrifuged for 10 min at ~1000 rpm to pellet large tissue chunks. For the unfiltered raw homogenate treatment group (exposed), the supernatant was used as inoculum. This inoculum was subsequently confirmed to contain *Vibrio pectenicida* , a strain of which was recently demonstrated to cause SSWD in sunflower sea stars (Prentice et al. 2025). For the control treatment group, a portion of supernatant was boiled for 10 min, before being cooled on ice and used as the heat‐killed inoculum. The adults were injected with the respective treatment inoculum (400 μL) using a 26G needle at four sites: three “armpits” (an intersection at the base of two arms), and one at a ~45° angle on the disc surface near the madreporite, with each injection being approximately 100 μL. The experiment ran for 14 days, during which sea stars were observed twice per day. At each observation, any disease signs or anything straying from the “normal” appearance of a relaxed *P. helianthoides* were noted. The sea stars were also photographed once per day. We used the following objective criteria to classify the gross condition of each sea star: a sea star was scored as grossly “normal” if it had even turgor, full attachment to the tank, and no arm twisting or lesions; as “arm twisting” if one or more arms curled or twisted away from the substrate; as “arm autotomy” if one or more arms detached from the central disc; and as deceased when tube feet no longer moved, once the central disc was no longer attached to the substrate, and all or most arms were autotomized. For photograph examples, see: Prentice et al. (2025).

**TABLE 1:** Experimental treatments and sample sizes for Adult SSWD challenge and Age Class SSWD Challenge experiments.

Experiment	Treatment group	Individuals per treatment	Proportion mortality	Number of individuals sequenced with RNAseq
Adult SSWD Challenge	Exposed	8	0.75	8
	Control	8	0	8
Age Class SSWD Challenge	Adult exposed	9	1	3
	Juvenile exposed	10	1	3
	Adult control	15	0	3
	Juvenile control	15	0	3

For the Age Class SSWD experiment, 24 adult and 25 juvenile quarantined *P. helianthoides* were selected and paired based on size. Individuals that were < 20 cm diameter were considered juveniles and > 30 cm diameter, adults, based on estimated minimum size for reproduction (Jason Hodin pers. comm.; Gehman et al. 2025). Juveniles ranged in diameter from 8 to 14 cm, and adults ranged in size from 32 to 44 cm in diameter (Data S1). One individual from each pair was randomly assigned to two treatment groups: (1) exposed (*n* = 9 adults, *n* = 10 juveniles), which were injected with unfiltered raw tissue homogenate made from a wasting adult *P. helianthoides* , or (2) control (*n* = 15 adults, *n* = 15 juveniles), which were injected with heat‐killed tissue homogenate made from the same wasting adult *P. helianthoides* (Table 1). Homogenates were prepared as described above for the Adult SSWD Challenge experiment, and *Vibrio pectenicida* was subsequently identified to be disproportionately present in this inoculum (Prentice et al. 2025). Adults were injected with their respective treatment inoculum (400 μL) using a 26G needle at four sites: three “armpits”, and one at a ~45° angle on the central disc surface near the madreporite, with each injection being approximately 100 μL. The average size of the juveniles was 10.6 cm diameter at collection time, and for adults it was 37.6 cm diameter. It was not possible to quantify the amount of pathogen(s) present in the inoculum to determine a specific dosage, so juveniles were injected with half the amount of inoculum (200 μL) as adults using a 29G needle at the same four sites, each injection being approximately 50 μL. These volumes were chosen in order to approximate equivalent ratios of host coelomic fluid to inoculum. The experiment ran for 18 days, and the sea stars were observed twice per day. At each observation, any disease signs or anything straying from the “normal” appearance of a relaxed *P. helianthoides* was noted and the sea stars were photographed twice daily, see Prentice et al. (2025) for photograph examples of disease signs.

For both experiments, when an exposed sea star autotomized an arm as the disease progressed (on average at day 10 post‐exposure), the sea star and its control pair were sampled nonlethally for coelomocytes. To do so, coelomic fluid was collected using a 26G needle and 1 mL syringe for adults, or a 29G needle and 300 μL syringe for juveniles, and deposited into a 1.7 mL microcentrifuge tube that was placed on ice. The amount of coelomic fluid retrieved during sampling varied with each sea star but was approximately 300–500 μL, and there were a maximum of 5 collection attempts (needle pricking) per sea star per sampling. To concentrate the coelomocyte cells, the samples of coelomic fluid were centrifuged for 5 min at 1200 rpm, and the supernatant was removed. The pelleted coelomocytes were preserved in RNAlater (500 μL) (ThermoFisher Scientific) and immediately stored at −80°C until RNA extraction.

### 2.3 Survival Comparison Between Adults and Juveniles

Differences in days to mortality between juveniles (diameter < 20 cm) and adults (diameter > 30 cm) were investigated, using an ANOVA to compare the mean days to mortality between the two age groups. Mortality in our work is defined as the time point when most or all arms are autotomized and the central disc is no longer attached to the tank.

### 2.4 RNAseq Alignment and Annotation

RNA from the coelomocyte samples was extracted using the Zymo Quick DNA/RNA Microprep Plus Kit (Zymo Research, cat: D7005) for cells stored in RNAlater according to the manufacturer's protocol. Extracted RNA (1 μL) was quantified using a Qubit 3.0 with the Qubit RNA HS Kit (Invitrogen, cat: Q32855). Samples for RNAseq were selected, balancing the number of samples sequenced from each treatment group and the amount and quality of RNA retrieved post‐extraction. RNA sequencing of 16 sea stars from the Adult SSWD Challenge and 12 sea stars from the Age Class SSWD Challenge (Table 1) was carried out by Azenta Life Sciences using an Illumina NovaSeq (150 bp paired end reads).

Raw sequence data were quality checked using FastQC (v0.11.9; Andrews 2010) and summarized with MultiQC (Ewels et al. 2016), pre and post trimming. Data was trimmed using fastp (v0.20.0; Chen et al. 2018) with the “–detect_adapter_for_pe” setting. Sequences were aligned to the *P. helianthoides* genome (Schiebelhut et al. 2024) using hisat2 (v2.2.1; Kim et al. 2019) and stringtie (v2.2.1; Pertea et al. 2015). Genes were annotated with SwissProt and gene ontology IDs by performing a BLASTx search (Altschul et al. 1990; Camacho et al. 2009) against the Uniprot Swissprot database. Gene ontologies were categorized into Biological Process GOslims using the Gene Ontology Consortium goslim_generic.obo (downloaded 20241030). Code and associated genomic data files are available at https://doi.org/10.5281/zenodo.14728718.

### 2.5 Differential Gene Expression

Differential gene expression analyses were performed for each experiment independently, using the R package DESeq2 (Love et al. 2014). For the Age Class SSWD Challenge, additional differential gene expression analyses were performed using a multifactor approach, which evaluates the effect of a specified treatment group, in this case age, on gene expression. For this analysis, age was set as a categorical variable, either “adult” or “juvenile”, with the control group set as the reference group. Finally, the interaction between age and disease was assessed using a contrast function which then was used to create an interaction model between age and infection status (Ekiz 2024). Code and associated data files for these analyses are available at https://doi.org/10.5281/zenodo.14728718.

### 2.6 Functional Enrichment

From both experiments' differentially expressed gene (DEG) lists, enriched biological processes were identified using DAVID (Huang et al. 2009; Sherman et al. 2022). We defined DEGs as those with an adjusted *p*‐value of less than 0.05. DAVID identifies overrepresented functional terms in a gene list (i.e., DEGs) to provide insight into physiological processes impacted under experimental conditions. The gene lists used for DAVID were the uniprot accession IDs from the DEG lists, with a background of the uniprot accession IDs from the genome. Biological processes with Benjamini–Hochberg procedure corrected *p*‐values of less than 0.05 were considered significantly enriched processes.

The overlap of DEGs across both experiments was also analyzed. The enriched biological processes that were shared between the experiments were identified using DAVID on the overlap of DEGs list. Biological processes with Benjamini–Hochberg procedure corrected *p*‐values of less than 0.05 were considered significantly enriched processes. Code and associated data files for these analyses are available at https://doi.org/10.5281/zenodo.14728718.

## 3 Results

### 3.1 Disease Progression and Survival

**FIGURE 1:**
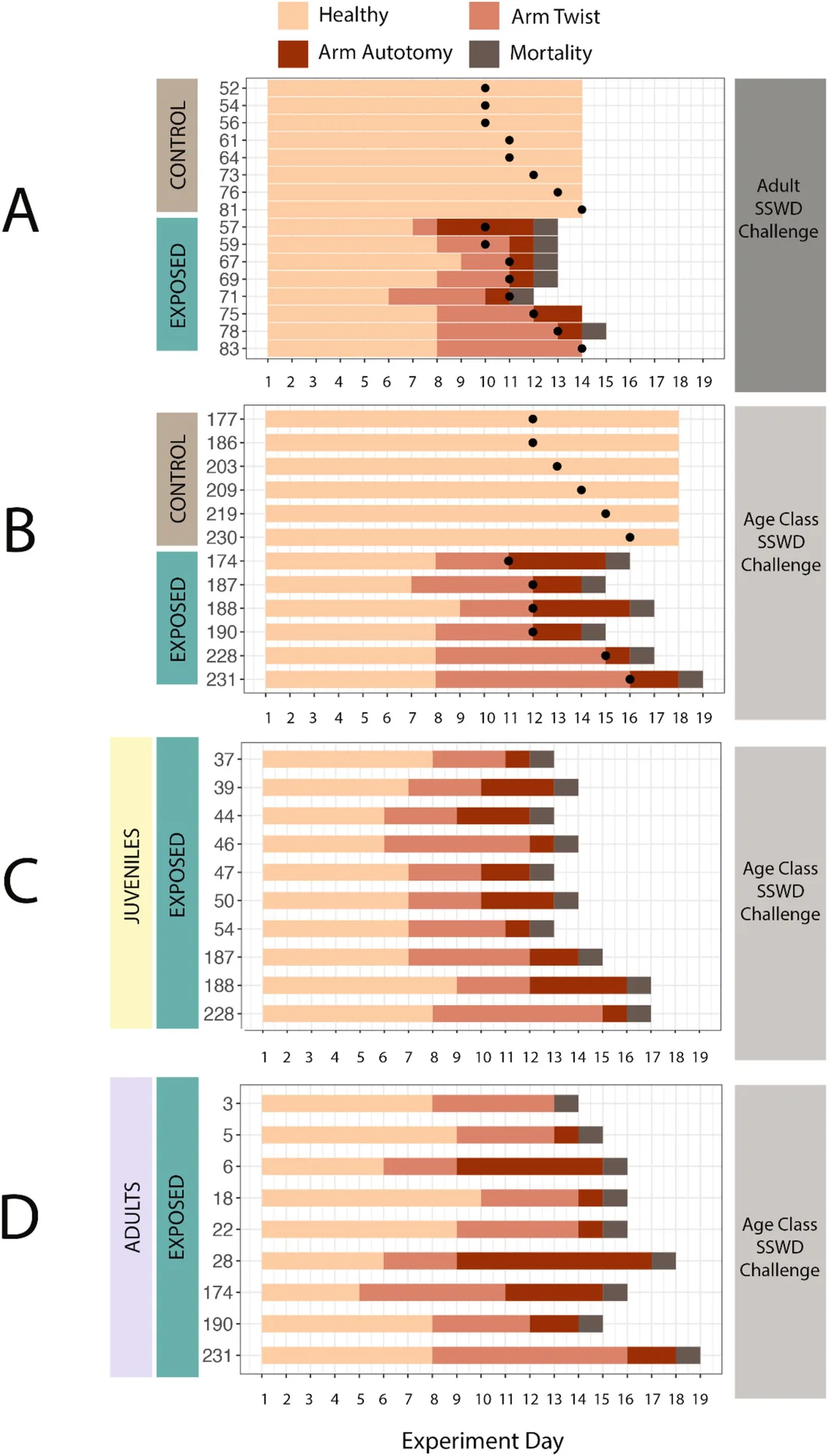
Disease sign progression of *P. helianthoides* in the Adult SSWD Challenge (Panel A), of the sea stars in the Age Class SSWD Challenge that have RNAseq data (Panel B), the juveniles in the exposed treatment from the Age Class SSWD Challenge (Panel C), and the adults in the exposed treatment from the Age Class SSWD Challenge (Panel D). Each row represents an individual sea star and corresponds to the sea star's RNAseq library ID number. Time points in light orange are when the sea stars are grossly healthy in appearance; pink time points are when the sea stars are twisting arms; red are when the sea stars have started arm autotomy; and if the timeline ends in dark gray, the sea star died as a result of the exposure treatment. Black dots on each individual timeline in panels (A, B) reflect coelomocyte sampling timepoints that were sequenced for RNA‐seq. Due to limitations in sample volume, RNA was not able to be extracted from every time point, hence the unpaired sampling times for sequencing.

**FIGURE 2:**
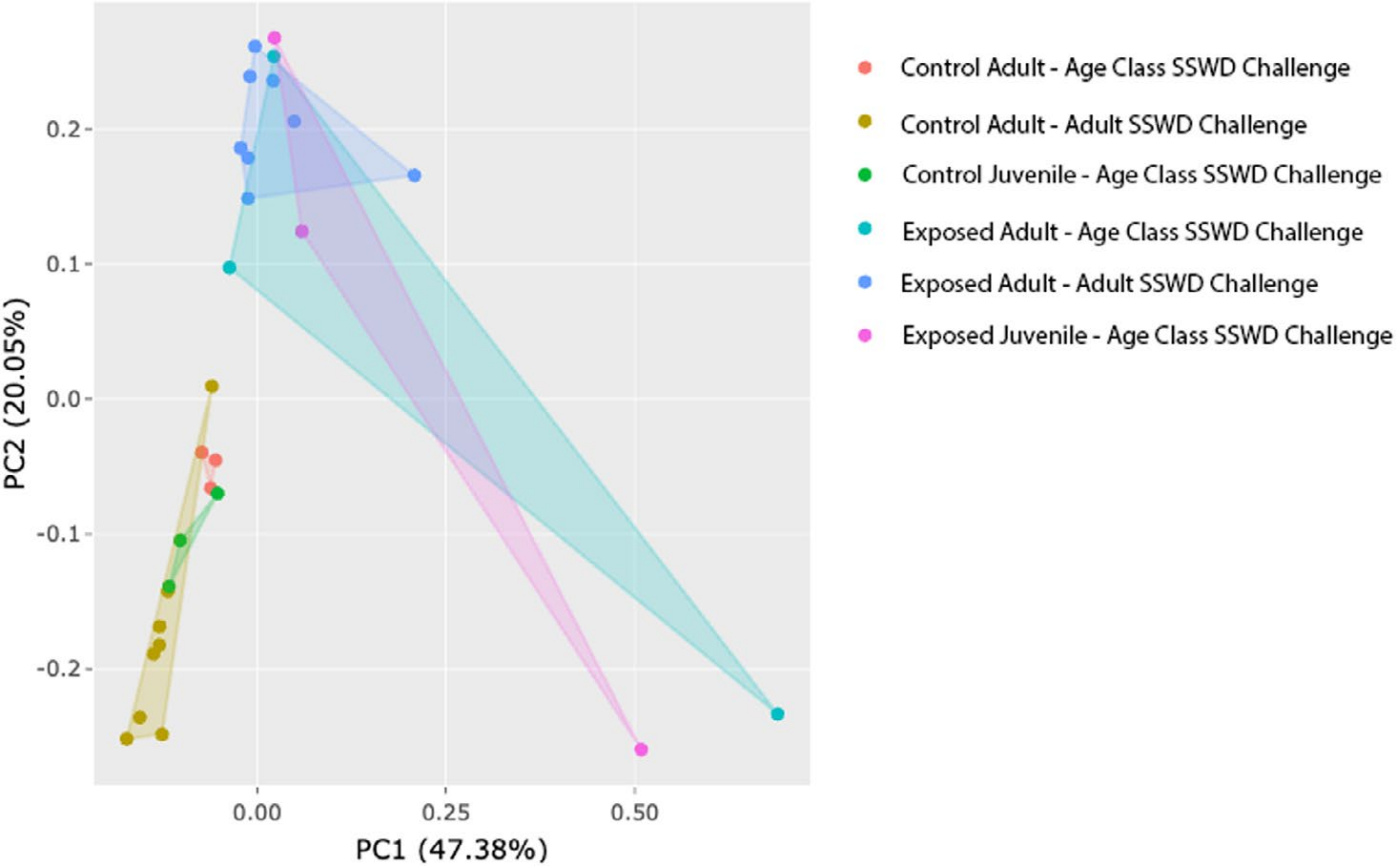
RNAseq counts post genome alignment plotted in a PCA. Points are color‐coded by treatment group and experiment. Pink are control adult *P. helianthiodes* from the Age Class SSWD Challenge; yellow are the control adult *P. helianthoides* from the Adult SSWD Challenge; green are the control juvenile *P. helianthiodes* from the Age Class SSWD Challenge; light blue/green are the exposed adult *P. helianthoides* from the Age Class SSWD Challenge; blue are the exposed adult *P. helianthiodes* from the Adult SSWD Challenge; and purple are the exposed juvenile *P. helianthoides* from the Age Class SSWD Challenge. The control groups (across experiments and age classes) are clustered together, and the exposed groups (across experiments and age classes) are clustered together, with no overlap between the exposed and control groups across both experiments and age classes.

Consistently observed disease signs in *P. helianthoides* exposed to SSWD included arm twisting, arm loss (or autotomy), and mortality (Figures 1A and 2; Prentice et al. 2025). For the Adult SSWD Challenge, all 8 control sea stars remained free of disease signs throughout the 14 day experiment. Mortality was observed in 6 of the 8 exposed stars, with the remaining 2 showing disease signs (arm twisting in one individual and arm twisting followed by arm autotomy in the other) by day 14 (Figure 1A).

For the Age Class SSWD Challenge, mortality was observed in all exposed stars (adults: *n* = 9; juveniles: *n* = 10) whereas all control sea stars (adults: *n* = 15; juveniles: *n* = 15) remained disease‐sign free throughout the 18 day experiment (Table 1, Figure 1C,D). The 3 exposed adult sea stars selected for sequencing all dropped at least one arm between days 11 and 16 (mean 13 days to arm loss), and the 3 juvenile exposed sea stars selected for sequencing all dropped at least one arm between days 12 and 15 (mean 13 days to arm loss) (Figure 1B). The time from exposure to mortality was significantly different between juveniles and adults (ANOVA, *p* = 0.02, sum of squares = 15.54), with juvenile mean mortality at 12.3 days (standard error ±0.495) preceding adult mean mortality at 14.1 days (standard error ±0.512).

### 3.2 RNAseq Alignment and Annotation

There were 23,464 genes that had RNAseq alignment to the *P. helianthoides* genome. There were 17,678 genes annotated with BLASTx.

RNAseq libraries from both experiments aligned to the *P. helianthoides* genome at rates ranging from 63.35% to 81.64% for 27 of the 28 libraries, with one library aligning at a rate of 46.60% (Data S1). Average alignment rates for all libraries was 76.84% ± standard error 1.32%. Transcript counts following alignment were plotted in a PCA (Figure 2). This plot showed clustering of samples by treatment group, but not age class, suggesting that the variation in transcriptomic response across *P. helianthoides* samples was largely driven by treatment (i.e., whether the individual was exposed to raw unfiltered vs. heat‐killed tissue homogenate inoculum) rather than age.

### 3.3 Differential Gene Expression

For the Adult SSWD Challenge, we identified 6938 differentially expressed genes (DEGs) between control and exposed treatment groups (Figure S1a, Data S2). Of those DEGs, 4003 were over‐expressed in exposed sea stars compared to controls.

For the Age Class SSWD Challenge, we identified 6237 DEGs between control and exposed treatment groups (Figure S1b, Data S3). This comparison shows high‐level differences, focusing on exposure status comparison regardless of age. Of those DEGs, 3628 were over‐expressed in exposed compared to control sea stars. In the multifactor analysis with both treatment and age as factors we did not find a substantial difference in DEGs (6202) when controlling for age and comparing condition (SSWD‐exposure vs. control) (design = ~age + condition; Data S4). A contrast on the previous DEG analysis was performed, creating a linear combination of estimated log2 fold changes, resulting in a list of 82 differentially expressed genes (Data S5). This comparison highlights genes that are the inverse comparison of the previous DEG list—the DEGs when controlling for condition and comparing age (design = ~condition + age). Additionally, the interaction between age and treatment yielded 140 DEGs (Data S8). The interaction analysis tests whether the effects of condition (SSWD‐exposure or control) change with age (design = ~condition + age + condition:age). These analyses from the Age Class SSWD Challenge exclude the adults from the Adult SSWD Challenge.

There are 4114 DEGs (45.4% of all total DEGs identified across both experiments) shared between the two experiments (Data S6), with 99.7% possessing the same expression directionality, and fall into 70 parental gene ontology (GOSlim) categories (Data S7).

Of the 23,464 genes aligned to the genome and characterized as part of this study, 30% and 27% were differentially expressed in the Adult SSWD Challenge and Age Class SSWD Challenge respectively. Differentially expressed genes identified in both experiments (4114) represent 17.5% of the genes that aligned to the genome.

### 3.4 Functional Enrichment

**FIGURE 3:**
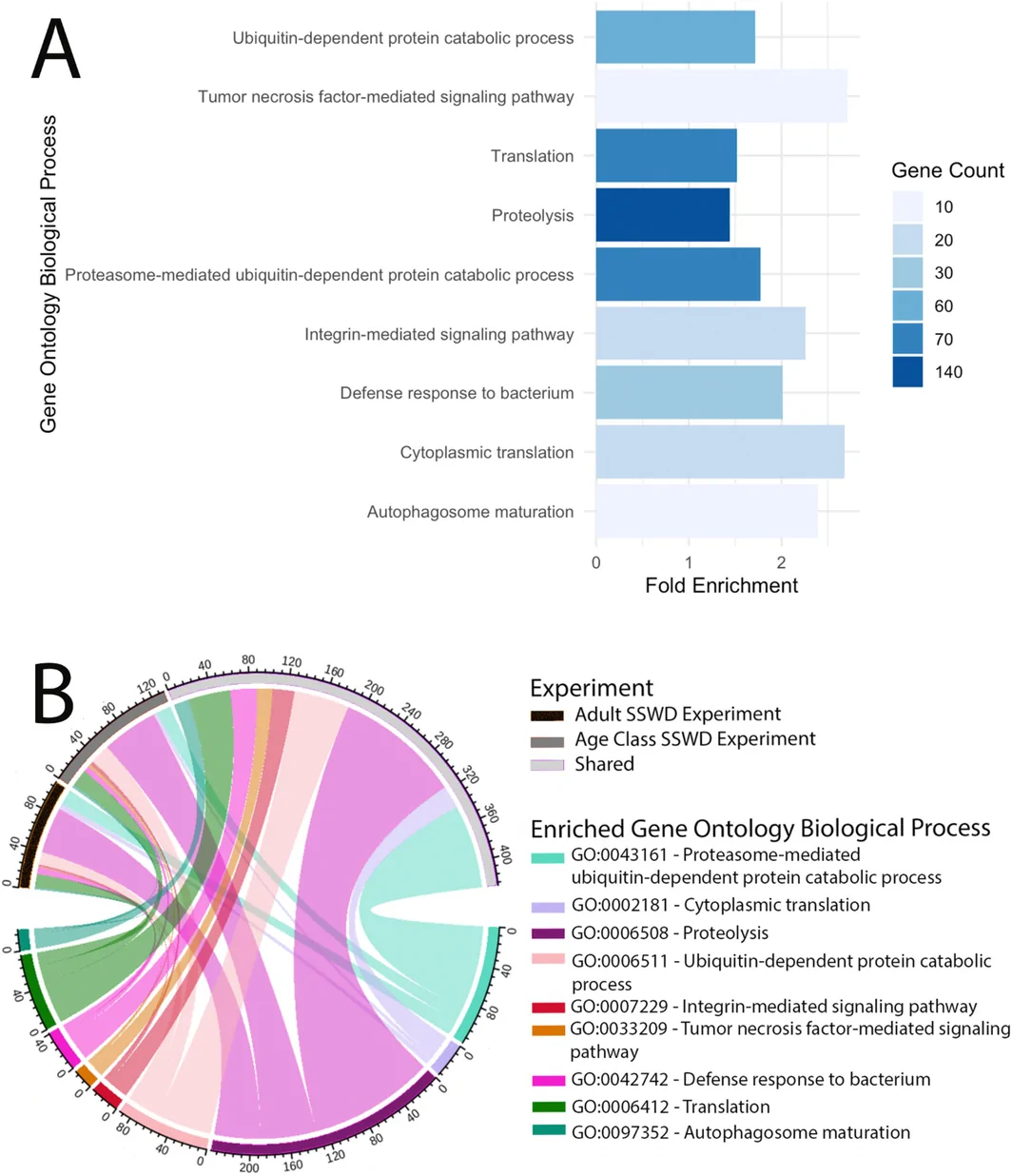
(A) The fold enrichment of significantly enriched (Benjamini–Hochberg corrected *p*‐value < 0.05) biological processes in exposed *P. helianthoides* . Bars are color‐coded by counts of DEGs annotated by the term. Functional enrichment was carried out on the common 4114 DEGs found in both experiments. (B) DEGs associated with enriched biological processes unique to each experiment and those that are shared by both experiments, and corresponding biological processes. The top portion represents DEGs grouped by experiment (black = Adult SSWD Experiment, dark gray = Age Class SSWD Experiment, and light gray = DEGs shared between the two experiments). The bottom portion of the plot represents the 9 significantly enriched biological processes. The color of the chords connecting the two portions matches the biological processes. The numbers around the plot represent the number of DEGs associated with the experiments (top semicircle) and the number of DEGs associated with the GO term (bottom semicircle). This diagram was made using the circlize R package (Gu et al. 2014).

Functional gene enrichment analysis was also carried out on several DEG lists. For the list of 140 DEGs that resulted from the interaction between age and SSWD‐exposure status (Data S8), no enrichment was identified. There were 51 GOSlim terms annotated to the 140 DEGs (Data S9). Some of the top GOSlim biological processes identified were related to development, signaling, differentiation, and stress and metabolism regulation. For the 4114 DEGs found in both experiments, functional enrichment analysis resulted in 211 over‐represented biological processes (Data S10). Of those, 9 terms had a Benjamini–Hochberg corrected *p*‐value < 0.05 (Figure 3A, Data S11). Predominant biological processes included ubiquitin‐dependent protein catabolic processes; tumor necrosis factor‐mediated signaling pathway; proteolysis; proteasome‐mediated ubiquitin‐dependent protein catabolic process; and defense response to bacterium. There are 488 differentially expressed genes that contributed to those 9 significantly enriched biological processes (Figure 3B, Data S12). Genes contributing to key innate immune responses in *P. helianthoides* exposed to tissue homogenate from SSWD‐affected *P. helianthoides* are listed in Table 2.

**TABLE 2:** Key genes contributing to innate immune response functions in *P. helianthoides* exposed to tissue homogenate from SSWD‐affected *P. helianthoides* .

Functional group	Genes
Apoptosis	RIPK1; JNK; TNR19
NF‐kB signaling pathway	TRAF5; TN13B; TRAF2; TRAF4; TRAF5; TRAF6; BIRC2; IKKA; RNF31
MAPK signaling	TRAF5; TRAF2; TRAF6
Inflammation	TRAF5; ADA17; TRAF2; TRAF4; TRAF6; JNK
Toll‐like receptor signaling pathway	MYD88

## 4 Discussion

This study aimed to characterize and compare the immune response of juvenile and adult *P. helianthoides* exposed to SSWD via injection with tissue homogenate from a wasting adult *P. helianthoides* . Both experiments successfully utilized an unfiltered tissue homogenate that contained a live, replicating microbial community (including, as subsequently confirmed, *V. pectenicida* ) and elicited disease signs not observed in the heat‐killed control group. Further, both experiments had consistent outcomes, where all control treatment sea stars remained disease‐sign free and all exposed sea stars progressed through characteristic SSWD signs. We identified a core set of genes that drove the immune response in wasting *P. helianthoides* , indeed the majority of the differentially expressed genes identified were shared across two independent experiments. Interestingly, adult and juvenile *P. helianthoides* exposed in our second experiment were equally susceptible to SSWD; however there were differences in disease progression timing and some DEGs identified that were driven specifically by age. These results contrast with previous field observations that hypothesized that juveniles are resistant to SSWD because they are not commonly seen wasting in the field. Instead, our work suggests that other explanations underlie the limited observation of wasting juveniles in the field.

While we did not observe a difference in resistance to SSWD in terms of mortality, we did detect a difference in disease progression and gene expression between juvenile and adult *P. helianthoides* . In our Age Class SSWD Challenge, all adult and juvenile *P. helianthoides* exposed to raw tissue homogenate died; however, juveniles died at a significantly faster rate (12.3 days to mortality compared to 14.1 days to mortality for the adults). This difference could be driven by a possible variation in the pathogen dose (likely *Vibrio pectenicida* ) in the exposure inoculum between adults and juveniles. Pathogen abundance was not quantified in the inoculum, and thus the volume difference does not necessarily directly relate to dose; however, the tissue homogenate inocula was well‐mixed, and the same inocula was used to expose both adults and juveniles. In a similar challenge experiment, Pacific oysters exposed to pathogenic *Vibrio* bacteria showed pathogen concentration in host tissue was incongruous with differences in survival curves between juvenile and adult age classes (Green et al. 2016). Further work with a known dose of pathogen, possible now that *V. pectenicida* is known to be a causative agent of a SSWD‐like condition in *P. helianthoides* , could more explicitly evaluate this question. Alternatively, juveniles could die at a faster rate following exposure to SSWD, and that could explain the lack of observations of diseased animals in the field, as has been shown for *Pisaster ochraceus* (Eisenlord et al. 2016). An additional difference noted in this study is that there were 140 DEGs identified as being expressed as a result of the interaction between SSWD exposure and age. In other words, there were 140 DEGs related to SSWD exposure that change with age class. Functional enrichment analysis did not identify any DEGs as being enriched, but in looking at the annotations of the DEGs (92 of the 140 have annotations), the top GOSlim biological processes identified are related to development, signaling, differentiation, and stress and metabolism regulation. These transcriptomic differences could be driving the difference observed in disease progression, or could be related to the juveniles responding to a different dose relative to body size than the adults. Additionally, juveniles and adults differ in a number of physiological and ecological respects that could shape responses, including a higher surface‐area‐to‐volume ratio in juveniles, possible differences in physiological tolerances to environmental stressors and toxicants, and ongoing developmental and growth‐related processes.

The transcriptomic response of the sea stars in this study has some similarities and differences to that of a previous study, Fuess et al. (2015). Many of the differentially expressed genes shared between the two experiments in this study were related to the immune response, including genes involved in the tumor necrosis factor‐mediated signaling pathway and proteolysis. This transcriptional response is consistent with previous work characterizing SSWD in *P. helianthoides* where Fuess et al. (2015) reported differential expression of genes involved in the activation of immune response. There are, however, some differences in the overall transcriptomic response between this study and Fuess et al. (2015) that could be attributed to several factors including a difference in the inoculum used for exposures. Specifically, the sea stars in this study were exposed to unfiltered tissue homogenate and thus were responding to a wider range of potential microorganisms, whereas Fuess et al. (2015) exposed sea stars to 0.22 μm filtered inoculum, thus excluding most prokaryotic and eukaryotic microorganisms. Other differences include differences in source populations of the collected sea stars, sample size, and genomic resources used to characterize and contrast the immune responses of different treatment groups (i.e., our current work was able to utilize the recently published *P. helianthoides* genome; Schiebelhut et al. 2024). A formal homology‐aware re‐analysis of the Fuess et al. (2015) libraries against the now‐published *P. helianthoides* genome (Schiebelhut et al. 2024) is a next step that could potentially recontextualize the findings from that work with the current available resources.

The immune response of *P. helianthoides* is characterized by genes associated with inflammation, apoptosis, and cytokine production, all key players in innate immunity. The tumor necrosis factor‐mediated signaling pathway plays key functional roles in inflammation and programmed cell death (Holbrook et al. 2019). We note that apoptotic signaling and the TNF pathway are also implicated in tissue remodeling and limb autotomy in echinoderms (Fuess et al. 2015); thus the up‐regulation of TNR19, RIPK1, JNK, and TRAF‐family genes observed here may reflect a combination of pathogen‐driven inflammatory signaling and the apoptotic processes that accompany arm autotomy, the gross sign at which we sampled. Specific differentially expressed genes associated with the process are involved in apoptosis (RIPK1, JNK, TNR19), NF‐kB signaling pathway (TRAF5, TN13B, TRAF2, TRAF4, TRAF6, BIRC2, IKKA, and RNF31), MAPK signaling (TRAF5, TRAF2 and TRAF6), and inflammation (TRAF5, ADA17, TRAF2, TRAF4, TRAF6, and JNK). Apoptosis is an important process in immunity as host cell death in response to infection can be a mechanism to limit pathogen replication, by killing a self‐cell that has been infected (Alberts et al. 2002). MAPK signaling is involved in innate immunity processes such as inflammation and promoting cytokine production (Arthur and Ley 2013). The JNK protein and associated pathway proteins have been found to be increased in hemocytes and gills in mussels (*Perna viridis*) 3–6 h after exposure to the dinoflagellate, *Prorocentrum lima* , and the toxin it produces, DSP (Diarrhetic Shellfish Poisoning) (Lv et al. 2021), suggesting an important role in toxin and immune response in invertebrates.

We found an important gene, MYD88, which encodes a Toll‐like receptor adapter known to recognize a broad range of microbial molecules. While MYD88 is consistently over‐represented in our analyses and supports an active immune response to the introduced inoculum, we note that MYD88 is not strictly bacterium‐specific and has been shown to recognize eukaryotic agents as well, so its over‐expression here should be interpreted as a general microbial‐defense response that is consistent with, but not solely diagnostic of, infection by *V. pectenicida* . As we now know that *V. pectenicida* was present within the experimental inoculum (Prentice et al. 2025), this supports the hypothesis that the stars are mounting an immune response to fight this microbial (including bacterial) infection. Genes contributing to this process include those involved in key functional processes such as NF‐κB signaling, protein degradation, and RNA processing and translation. For example, differentially expressed genes annotated as MYD88 (Data S6) showed elevated expression in exposed sea stars. MYD88 has been described as being a key gene in toll‐like receptor signaling pathways in *Haliotis diversicolor* (Gong et al. 2023) and *Crassostrea gigas* (Fan et al. 2022) following exposure to bacteria, specifically within the genus *Vibrio*. Further, NF‐kB signaling is a process involved in immune cell differentiation and inflammation (Liu et al. 2017), suggesting that the sea stars are actively increasing their immune cell counts and, through proliferation, subsequently driving an inflammatory response to bacteria.

Increased transcriptional activity associated with protein degradation is consistent with the role of proteasome activity as a part of the bacterial immune response (Kammerl and Meiners 2016). Although all stars exposed to SSWD in these exposure trials died, the fact that their immune systems were responding to bacterial and other microbial components introduced via the unfiltered homogenate, including *V. pectenicida* , suggests that *P. helianthoides* is fighting an infection. Further work could look into dose response of *V. pectenicida* , and taken with this study's findings of immune response, there could be the potential for successful fighting off of infection at lower doses. The increased protein degradation could be indicative of processing of damaged host proteins resulting from bacterial proteases acting to counteract the host immune response (Potempa and Pike 2009). Genes previously reported as involved in ubiquitination and bacterial protein degradation that show up in our dataset include RNF31 and RGLG4, which encode for E3 ubiquitin ligases. E3 ubiquitin ligases identify and bind to targets for ubiquitination, which is a process that removes damaged proteins (Guo and Tadi 2020; Jeong et al. 2023). Both genes were more highly expressed in exposed sea stars in both experiments. This could point to protein degradation as being a component of disease progression and mortality. There were also several genes associated with defense to bacterium involved in RNA processing and translation such as the RNA helicase DHX15 (a sensor that responds to both bacterial and viral nucleic acid patterns; its over‐expression is consistent with a broader antimicrobial nucleic acid sensing response rather than evidence of a strictly bacterial response). It is likely this is reflective of the role of RNA metabolism and translational control during the immune response.

Marine disease ecology is an emerging field, and the cascading negative effects that SSWD has had on the kelp forest ecosystems that *P. helianthoides* help regulate demonstrates the importance of this field. With increasing occurrences of extreme environmental events (e.g., marine heat waves, ocean chemistry shifts), and increasing anthropogenic pressure, it is likely that there will continue to be marine disease outbreaks, and that they will have wide‐ranging and long‐term impacts. Studying the immune response of hosts impacted by disease using transcriptomics will help with identifying physiological traits of importance in relation to fighting off disease. In instances where species have varied resistance to the same disease, traits related to resistance can be identified through transcriptomics and targeted in conservation breeding. Increasing the body of work that studies these systems will contribute to the tools used to understand, study, and mitigate disease outbreaks for the present and future.

## 5 Conclusions

Juvenile *P. helianthoides* are susceptible to sea star wasting disease. The considerable transcriptional response overlap between the two experiments described in this study indicates that there is a core set of genes that relate to immune response in wasting sunflower sea stars. Biological processes enriched across the two experiments (e.g., defense response to bacterium, tumor necrosis factor‐mediated signaling pathway), indicate that *P. helianthoides* is actively fighting off infection by microbes, including bacteria.

## Author Contributions

**Grace Crandall:** conceptualization, data curation, formal analysis, investigation, methodology, visualization, writing – original draft, writing – review and editing. **Alyssa‐Lois M. Gehman:** conceptualization, data curation, funding acquisition, investigation, methodology, supervision, writing – review and editing. **Katherine Davis:** conceptualization, data curation, investigation, writing – review and editing. **Melanie B. Prentice:** data curation, writing – review and editing. **Drew Harvell:** conceptualization, funding acquisition, supervision, writing – review and editing. **Samuel J. White:** resources, writing – review and editing. **Paul Hershberger:** resources, supervision, writing – review and editing. **Steven Roberts:** resources, supervision, writing – review and editing.

## Funding

This work was supported by Tula Foundation and Nature Conservancy (P119034).

## Conflicts of Interest

The authors declare no conflicts of interest.

## Supporting information


**Figure S1:** PCA of the differentially expressed RNAseq data from the libraries from the Adult SSWD Challenge (a) and the Age Class SSWD Challenge (b).
**Data S1:** Metadata for all RNAseq libraries with alignment rates.
**Data S2:** List of differentially expressed genes from the Adult SSWD Challenge experiment comparing the SSWD‐exposed and control treatments. DEGs are annotated with gene ontology.
**Data S3:** List of differentially expressed genes from the Age Class SSWD Challenge experiment. This list is from combining the adults and juveniles and comparing the controls to the SSWD‐exposed *P. helianthoides* . DEGs are annotated with gene ontology.
**Data S4:** List of differentially expressed genes from the Age Class SSWD Challenge experiment. This list is from controlling for age and comparing condition (SSWD‐exposure vs. control; design = ~age + condition). DEGs are annotated with gene ontology.
**Data S5:** List of differentially expressed genes from the Age Class SSWD Challenge experiment. This list is from a comparison that highlights genes that are the inverse comparison of the DEG list in Data S4. These are the DEGs when controlling for condition and comparing age (design = ~condition + age).
**Data S6:** This is the list of differentially expressed genes that are shared between the two experiments. This list is from finding what's shared between Data S2 and S3. The DEGs are annotated with gene ontology.
**Data S7:** This is a list of the counts of Gene Ontology Slim categories from the list of differentially expressed genes that are shared between the two experiments.
**Data S8:** This is a list of differentially expressed genes from the Age Class SSWD Challenge that are from the interaction of age and SSWD‐exposure. The interaction analysis tests whether the effects of condition (SSWD‐exposure or control) change with age (design = ~condition + age + condition:age). DEGs are annotated with gene ontology.
**Data S9:** This is a list of the counts of Gene Ontology Slim categories from the list of differentially expressed genes that are from the interaction of age and SSWD‐exposure (Data S8).
**Data S10:** DAVID enrichment output from the list of differentially expressed genes that are shared between the two experiments (Data S6).
**Data S11:** List of DAVID enriched output terms that have a Benjamini–Hochberg *p*‐value of less than 0.05.
**Data S12:** The list of differentially expressed genes that contribute to the significantly enriched terms from the DAVID output (Data S11).

## Data Availability

Code and associated data files are available at https://doi.org/10.5281/zenodo.14728718. Raw sequence files are available at NCBI, BioProject PRJNA1440140.

## References

[ece374379-bib-0001] Alberts, B. , A. Johnson , J. Lewis , et al. 2002. Innate Immunity. NCBI. https://www.ncbi.nlm.nih.gov/books/NBK26846/.

[ece374379-bib-0002] Altschul, S. F. , W. Gish , W. Miller , E. W. Myers , and D. J. Lipman . 1990. “Basic Local Alignment Search Tool.” Journal of Molecular Biology 215, no. 3: 403–410. 10.1016/S0022-2836(05)80360-2 2231712

[ece374379-bib-0003] Alvarez‐Filip, L. , F. J. González‐Barrios , E. Pérez‐Cervantes , A. Molina‐Hernández , and N. Estrada‐Saldívar . 2022. “Stony Coral Tissue Loss Disease Decimated Caribbean Coral Populations and Reshaped Reef Functionality.” Communications Biology 5, no. 1: 440. 10.1038/s42003-022-03398-6 PMC9184636 35681037

[ece374379-bib-0004] Andrews, S. 2010. “FASTQC: A Quality Control Tool for High Throughput Sequence Data.” http://www.bioinformatics.babraham.ac.uk/projects/fastqc/.

[ece374379-bib-0005] Aoki, L. , B. Yang , O. Graham , et al. 2023. “UAV High‐Resolution Imaging and Disease Surveys Combine to Quantify Climate‐Related Decline in Seagrass Meadows.” Oceanography 36, no. 1: 38–39. 10.5670/oceanog.2023.s1.12.

[ece374379-bib-0006] Arthur, J. S. C. , and S. C. Ley . 2013. “Mitogen‐Activated Protein Kinases in Innate Immunity.” Nature Reviews. Immunology 13, no. 9: 679–692. 10.1038/nri3495 23954936

[ece374379-bib-0007] Boettcher, K. J. , K. K. Geaghan , A. P. Maloy , and B. J. Barber . 2005. “ *Roseovarius crassostreae* sp. Nov., a Member of the Roseobacter Clade and the Apparent Cause of Juvenile Oyster Disease (JOD) in Cultured Eastern Oysters.” International Journal of Systematic and Evolutionary Microbiology 55, no. Pt 4: 1531–1537. 10.1099/ijs.0.63620-0 16014477

[ece374379-bib-0008] Bradley, P. W. , P. W. Snyder , and A. R. Blaustein . 2019. “Host Age Alters Amphibian Susceptibility to Batrachochytrium Dendrobatidis, an Emerging Infectious Fungal Pathogen.” PLoS One 14, no. 9: e0222181. 10.1371/journal.pone.0222181 PMC6730893 31491016

[ece374379-bib-0009] Burge, C. A. , C. Mark Eakin , C. S. Friedman , et al. 2014. “Climate Change Influences on Marine Infectious Diseases: Implications for Management and Society.” Annual Review of Marine Science 6, no. 1: 249–277. 10.1146/annurev-marine-010213-135029 23808894

[ece374379-bib-0010] Camacho, C. , G. Coulouris , V. Avagyan , et al. 2009. “BLAST+: Architecture and Applications.” BMC Bioinformatics 10, no. 1: 421. 10.1186/1471-2105-10-421 PMC2803857 20003500

[ece374379-bib-0011] Cardinaud, M. , N. M. Dheilly , S. Huchette , D. Moraga , and C. Paillard . 2015. “The Early Stages of the Immune Response of the European Abalone *Haliotis tuberculata* to a *Vibrio harveyi* Infection.” Developmental and Comparative Immunology 51, no. 2: 287–297. 10.1016/j.dci.2015.02.019 25766281

[ece374379-bib-0012] Chen, S. , Y. Zhou , Y. Chen , and J. Gu . 2018. “fastp: An Ultra‐Fast All‐in‐One FASTQ Preprocessor.” Bioinformatics 34, no. 17: i884–i890. 10.1093/bioinformatics/bty560 PMC6129281 30423086

[ece374379-bib-0013] Chiaramonte, M. , and R. Russo . 2015. “The Echinoderm Innate Humoral Immune Response.” Italian Journal of Zoology 82, no. 3: 300–308.

[ece374379-bib-0014] Dawson, M. N. , P. J. Duffin , M. Giakoumis , et al. 2023. “A Decade of Death and Other Dynamics: Deepening Perspectives on the Diversity and Distribution of Sea Stars and Wasting.” Biological Bulletin 244, no. 3: 143–163. 10.1086/727969 38457680

[ece374379-bib-0015] Eisenlord, M. E. , M. L. Groner , R. M. Yoshioka , et al. 2016. “Ochre Star Mortality During the 2014 Wasting Disease Epizootic: Role of Population Size Structure and Temperature.” Philosophical Transactions of the Royal Society of London. Series B, Biological Sciences 371, no. 1689: 20150212. 10.1098/rstb.2015.0212 PMC4760142 26880844

[ece374379-bib-0016] Ekiz, A. 2024. “A Guide to Designs and Contrasts in DESeq2.” https://www.atakanekiz.com/technical/a‐guide‐to‐designs‐and‐contrasts‐in‐DESeq2/.

[ece374379-bib-0017] Ewels, P. , M. Magnusson , S. Lundin , and M. Käller . 2016. “MultiQC: Summarize Analysis Results for Multiple Tools and Samples in a Single Report.” Bioinformatics 32, no. 19: 3047–3048. 10.1093/bioinformatics/btw354 PMC5039924 27312411

[ece374379-bib-0018] Fan, S. , W. Wang , J. Li , et al. 2022. “The Truncated MyD88s Negatively Regulates TLR2 Signal on Expression of IL17‐1 in Oyster *Crassostrea gigas* .” Developmental and Comparative Immunology 133, no. 104446: 104446. 10.1016/j.dci.2022.104446 35569578

[ece374379-bib-0019] Fuess, L. E. , M. E. Eisenlord , C. J. Closek , et al. 2015. “Up in Arms: Immune and Nervous System Response to Sea Star Wasting Disease.” PLoS One 10, no. 7: e0133053. 10.1371/journal.pone.0133053 PMC4503460 26176852

[ece374379-bib-0020] Galloway, A. W. E. , S. A. Gravem , J. N. Kobelt , et al. 2023. “Sunflower Sea Star Predation on Urchins Can Facilitate Kelp Forest Recovery.” Proceedings of the Royal Society B: Biological Sciences 290, no. 1993: 20221897. 10.1098/rspb.2022.1897 PMC9943640 36809801

[ece374379-bib-0021] Gao, Q. , M. Liao , Y. Wang , et al. 2015. “Transcriptome Analysis and Discovery of Genes Involved in Immune Pathways From Coelomocytes of Sea Cucumber ( *Apostichopus japonicus* ) After *Vibrio splendidus* Challenge.” International Journal of Molecular Sciences 16, no. 7: 16347–16377. 10.3390/ijms160716347 PMC4519954 26193268

[ece374379-bib-0022] Gehman, A.‐L. M. , O. Pontier , T. Froese , et al. 2025. “Fjord Oceanographic Dynamics Provide Refuge for Critically Endangered *Pycnopodia helianthoides* .” Proceedings of the Royal Society B: Biological Sciences 292, no. 2044: 20242770. 10.1098/rspb.2024.2770 PMC11961252 40169020

[ece374379-bib-0023] Gong, X. , M. Li , L. Zhang , S. Huang , and G. Wang . 2023. “Identification and Functional Analysis of Myeloid Differentiation Factor 88 (MyD88) in Early Development of *Haliotis diversicolor* .” Fish & Shellfish Immunology 142: 109085. 10.1016/j.fsi.2023.109085 37722440

[ece374379-bib-0024] Gravem, S. A. , W. N. Heady , V. R. Saccomanno , et al. 2021. “*Pycnopodia helianthoides*. IUCN Red List of Threatened Species.” https://www.reefcheck.org/wp‐content/uploads/2021/10/Final_IUCNAssessment.pdf.

[ece374379-bib-0025] Green, T. J. , A. Vergnes , C. Montagnani , and J. de Lorgeril . 2016. “Distinct Immune Responses of Juvenile and Adult Oysters ( *Crassostrea gigas* ) to Viral and Bacterial Infections.” Veterinary Research 47, no. 1: 72. 10.1186/s13567-016-0356-7 PMC4955271 27439510

[ece374379-bib-0026] Gu, Z. , L. Gu , R. Eils , M. Schlesner , and B. Brors . 2014. “Circlize Implements and Enhances Circular Visualization in R.” Bioinformatics 30, no. 19: 2811–2812. 10.1093/bioinformatics/btu393 24930139

[ece374379-bib-0027] Guo, H. J. , and P. Tadi . 2020. “Biochemistry, Ubiquitination.” https://www.ncbi.nlm.nih.gov/books/NBK556052/. 32310512

[ece374379-bib-0028] Hamilton, S. L. , V. R. Saccomanno , W. N. Heady , et al. 2021. “Disease‐Driven Mass Mortality Event Leads to Widespread Extirpation and Variable Recovery Potential of a Marine Predator Across the Eastern Pacific.” Proceedings of the Royal Society B: Biological Sciences 288, no. 1957: 20211195. 10.1098/rspb.2021.1195 PMC8385337 34428964

[ece374379-bib-0029] Harvell, C. D. , and J. B. Lamb . 2020. “Disease Outbreaks Can Threaten Marine Biodiversity.” In Marine Disease Ecology, 141–158. Oxford University Press.

[ece374379-bib-0030] Harvell, C. D. , D. Montecino‐Latorre , J. M. Caldwell , et al. 2019. “Disease Epidemic and a Marine Heat Wave Are Associated With the Continental‐Scale Collapse of a Pivotal Predator ( *Pycnopodia helianthoides* ).” Science Advances 5, no. 1: eaau7042. 10.1126/sciadv.aau7042 PMC6353623 30729157

[ece374379-bib-0031] He, Y. , A. Jouaux , S. E. Ford , et al. 2015. “Transcriptome Analysis Reveals Strong and Complex Antiviral Response in a Mollusc.” Fish & Shellfish Immunology 46, no. 1: 131–144. 10.1016/j.fsi.2015.05.023 26004318

[ece374379-bib-0032] Heady, W. N. , R. Beas‐Luna , M. N. Dawson , et al. 2022. Roadmap to Recovery for the Sunflower Sea Star ( *Pycnopodia helianthoides* ) Along the West Coast of North America. Nature Conservancy. http://paperpile.com/b/2Sma3r/cVSGb.

[ece374379-bib-0033] Holbrook, J. , S. Lara‐Reyna , H. Jarosz‐Griffiths , and M. McDermott . 2019. “Tumour Necrosis Factor Signalling in Health and Disease.” F1000Research 8: 111. 10.12688/f1000research.17023.1 PMC6352924 30755793

[ece374379-bib-0034] Huang, D. W. , B. T. Sherman , and R. A. Lempicki . 2009. “Systematic and Integrative Analysis of Large Gene Lists Using DAVID Bioinformatics Resources.” Nature Protocols 4, no. 1: 44–57. 10.1038/nprot.2008.211 19131956

[ece374379-bib-0035] Jeong, Y. , A.‐R. Oh , Y. H. Jung , H. Gi , Y. U. Kim , and K. Kim . 2023. “Targeting E3 Ubiquitin Ligases and Their Adaptors as a Therapeutic Strategy for Metabolic Diseases.” Experimental & Molecular Medicine 55, no. 10: 2097–2104. 10.1038/s12276-023-01087-w PMC10618535 37779139

[ece374379-bib-0036] Kammerl, I. E. , and S. Meiners . 2016. “Proteasome Function Shapes Innate and Adaptive Immune Responses.” American Journal of Physiology. Lung Cellular and Molecular Physiology 311, no. 2: L328–L336. 10.1152/ajplung.00156.2016 27343191

[ece374379-bib-0037] Kim, D. , J. M. Paggi , C. Park , C. Bennett , and S. L. Salzberg . 2019. “Graph‐Based Genome Alignment and Genotyping With HISAT2 and HISAT‐Genotype.” Nature Biotechnology 37, no. 8: 907–915. 10.1038/s41587-019-0201-4 PMC7605509 31375807

[ece374379-bib-0038] Krammer, F. , E. Hermann , and A. L. Rasmussen . 2025. “Highly Pathogenic Avian Influenza H5N1: History, Current Situation, and Outlook.” Journal of Virology 99, no. 4: e0220924. 10.1128/jvi.02209-24 PMC11998540 40145745

[ece374379-bib-0039] Lambert, C. , J. L. Nicolas , V. Cilia , and S. Corre . 1998. “ *Vibrio pectenicida* sp. nov., a Pathogen of Scallop ( *Pecten maximus* ) Larvae.” International Journal of Systematic Bacteriology 48 Pt 2, no. 2: 481–487. 10.1099/00207713-48-2-481 9731288

[ece374379-bib-0040] Li, H. , J. Zhao , Y. Li , et al. 2024. “Transcriptome Analysis Reveals Tissue‐Specific Responses of Mytilus Unguiculatus to *Vibrio alginolyticus* Infection.” Fish & Shellfish Immunology 144, no. 109301: 109301. 10.1016/j.fsi.2023.109301 38110106

[ece374379-bib-0041] Liu, T. , L. Zhang , D. Joo , and S.‐C. Sun . 2017. “NF‐κB Signaling in Inflammation.” Signal Transduction and Targeted Therapy 2: 1–9. 10.1038/sigtrans.2017.23 PMC5661633 29158945

[ece374379-bib-0042] Love, M. I. , W. Huber , and S. Anders . 2014. “Moderated Estimation of Fold Change and Dispersion for RNA‐Seq Data With DESeq2.” Genome Biology 15, no. 12: 550. 10.1186/s13059-014-0550-8 PMC4302049 25516281

[ece374379-bib-0043] Lv, J.‐J. , K.‐K. Yuan , M.‐Y. Lu , Z.‐B. He , H.‐Y. Li , and W.‐D. Yang . 2021. “Responses of JNK Signaling Pathway to the Toxic Dinoflagellate *Prorocentrum lima* in the Mussel Perna Viridis.” Ecotoxicology and Environmental Safety 227, no. 112905: 112905. 10.1016/j.ecoenv.2021.112905 34673413

[ece374379-bib-0044] Pertea, M. , G. M. Pertea , C. M. Antonescu , T.‐C. Chang , J. T. Mendell , and S. L. Salzberg . 2015. “StringTie Enables Improved Reconstruction of a Transcriptome From RNA‐Seq Reads.” Nature Biotechnology 33, no. 3: 290–295. 10.1038/nbt.3122 PMC4643835 25690850

[ece374379-bib-0045] Potempa, J. , and R. N. Pike . 2009. “Corruption of Innate Immunity by Bacterial Proteases.” Journal of Innate Immunity 1, no. 2: 70–87. 10.1159/000181144 PMC2743019 19756242

[ece374379-bib-0046] Prentice, M. , G. A. Crandall , A. M. Chan , et al. 2025. “ *Vibrio pectenicida* Strain FHCF‐3 Is a Causative Agent of Sea Star Wasting Disease.” Nature Ecology & Evolution 9: 1739–1751. 10.1038/s41559-025-02797-2 40760083

[ece374379-bib-0047] Rahman, M. , M. Sobur , M. Islam , et al. 2020. “Zoonotic Diseases: Etiology, Impact, and Control.” Microorganisms 8: 1405. 10.3390/microorganisms8091405. PMC7563794 32932606

[ece374379-bib-0048] Raymond, W. W. , J. S. Barber , M. N. Dethier , et al. 2022. “Assessment of the Impacts of an Unprecedented Heatwave on Intertidal Shellfish of the Salish Sea.” Ecology 103, no. 10: e3798. 10.1002/ecy.3798 PMC9786359 35726191

[ece374379-bib-0049] Renner, H. M. , J. F. Piatt , M. Renner , B. A. Drummond , J. S. Laufenberg , and J. K. Parrish . 2024. “Catastrophic and Persistent Loss of Common Murres After a Marine Heatwave.” Science 386, no. 6727: 1272–1276. 10.1126/science.adq4330 39666817

[ece374379-bib-0050] Ronza, P. , J. A. Álvarez‐Dios , D. Robledo , et al. 2021. “Blood Transcriptomics of Turbot *Scophthalmus maximus* : A Tool for Health Monitoring and Disease Studies.” Animals: An Open Access Journal From MDPI 11, no. 5: 1296. 10.3390/ani11051296 PMC8147184 33946507

[ece374379-bib-0051] Schiebelhut, L. M. , M. B. DeBiasse , L. Gabriel , K. J. Hoff , and M. N. Dawson . 2024. “A Reference Genome for Ecological Restoration of the Sunflower Sea Star, *Pycnopodia helianthoides* .” Journal of Heredity 115, no. 1: 86–93. 10.1093/jhered/esad054 PMC10838127 37738158

[ece374379-bib-0052] Sherman, B. T. , M. Hao , J. Qiu , et al. 2022. “DAVID: A Web Server for Functional Enrichment Analysis and Functional Annotation of Gene Lists (2021 Update).” Nucleic Acids Research 50, no. W1: W216–W221. 10.1093/nar/gkac194 PMC9252805 35325185

[ece374379-bib-0053] Smith, L. C. , V. Arizza , M. A. Barela Hudgell , et al. 2018. “Echinodermata: The Complex Immune System in Echinoderms.” In Advances in Comparative Immunology, 409–501. Springer International Publishing.

[ece374379-bib-0054] Yao, T. , J. Lu , C. Bai , Z. Xie , and L. Ye . 2021. “The Enhanced Immune Protection in Small Abalone *Haliotis diversicolor* Against a Secondary Infection With *Vibrio harveyi* .” Frontiers in Immunology 12: 685896. 10.3389/fimmu.2021.685896 PMC8290317 34295333

